# Giant cell tumor of bone in an eighteenth-century Italian mummy

**DOI:** 10.1007/s00428-021-03192-5

**Published:** 2021-08-30

**Authors:** Luca Ventura, Enrico Petrella, Sara Piciucchi, Elisabetta Cilli, Donata Luiselli, Robin N. M. Feeney, Mirko Traversari

**Affiliations:** 1 Division of Pathology, San Salvatore Hospital, L’Aquila, Italy; 2 Department of Biotechnological and Applied Clinical Sciences, University of L’Aquila, L’Aquila, Italy; 3 Department of Radiology, AUSL Romagna, Morgagni-Pierantoni City Hospital, Forlì, Italy; 4 Department of Cultural Heritage, University of Bologna, Ravenna, Italy; 5 Health Sciences Centre, UCD School of Medicine, University College Dublin, Belfield, Dublin, Ireland

**Keywords:** Osteoclastic giant cell–rich tumors, Giant cell tumor, Non-ossifying fibroma, Osteoclastoma, Benign fibrous histiocytoma, Fibrous cortical defect

## Abstract

Giant cell tumor (GCT) of the bone is a locally aggressive and rarely metastasizing neoplasm. It is composed of neoplastic mononuclear stromal cells with a monotonous appearance admixed with macrophages and osteoclast-like giant cells. In a small subset of cases, GCT is malignant. Terminology previously related to this entity, and which is no longer supported by the World Health Organization, includes osteoclastoma and benign fibrous histiocytoma (BFH). Giant cells occur in numerous other pathologic conditions of the bone, which accounts for the misrepresentation of these non-GCT tumors in the early literature. Non-ossifying fibroma (NOF), aneurysmal bone cyst, and chondroblastoma have been erroneously labeled GCT for this reason. A single description of an ancient GCT was reported by Brothwell and Sandison and subsequently mentioned by Aufderheide and Rodrìguez-Martìn who were astonished that more of these tumors had not been identified in archaeological cases. To the best of our knowledge, no other cases of ancient GCT have been cited in the paleopathology literature. The study of this type of neoplasm in antiquity can be used as a means to better understand its characteristics and behavior and to expand the depth of time of the etiology of these lesions. We report a case of GCT of the left femur observed following the total body CT imaging of a partially mummified adult female, dating to eighteenth century.

## Introduction

Giant cell tumor (GCT) of the bone is a locally aggressive and rarely metastasizing neoplasm. It is composed of neoplastic mononuclear stromal cells with a monotonous appearance admixed with macrophages and osteoclast-like giant cells [1]. In a small subset of cases, GCT is malignant.

Terminology previously related to this entity, and which is no longer supported by the World Health Organization, includes osteoclastoma and benign fibrous histiocytoma (BFH) [2–6]. Giant cells occur in numerous other pathologic conditions of the bone, which accounts for the misrepresentation of these non-GCT tumors in the early literature. Non-ossifying fibroma (NOF), aneurysmal bone cyst, and chondroblastoma have been erroneously labeled GCT for this reason [7]. Current concepts identify BFH as a heterogeneous group of lesions [8]. Lesions formerly designated as BFH are now regarded as GCTs when they are located in the pelvic bones or epiphyses in the mature skeleton, whereas those occurring in the metaphyses are considered to represent NOFs [5, 6, 9, 10]. A single description of an ancient GCT was reported by Brothwell and Sandison [11] and subsequently mentioned by Aufderheide and Rodrìguez-Martìn who were astonished that more of these tumors had not been identified in archaeological cases [4]. To the best of our knowledge, no other cases of ancient GCT have been cited in the paleopathology literature. The study of this type of neoplasm in antiquity can be used as a means to better understand its characteristics and behavior and to expand the depth of time of the etiology of these lesions.

We report a case of GCT of the left femur observed following the total body CT imaging of a partially mummified adult female, dating to eighteenth century.

## Archaeological context

**Fig. 1:**
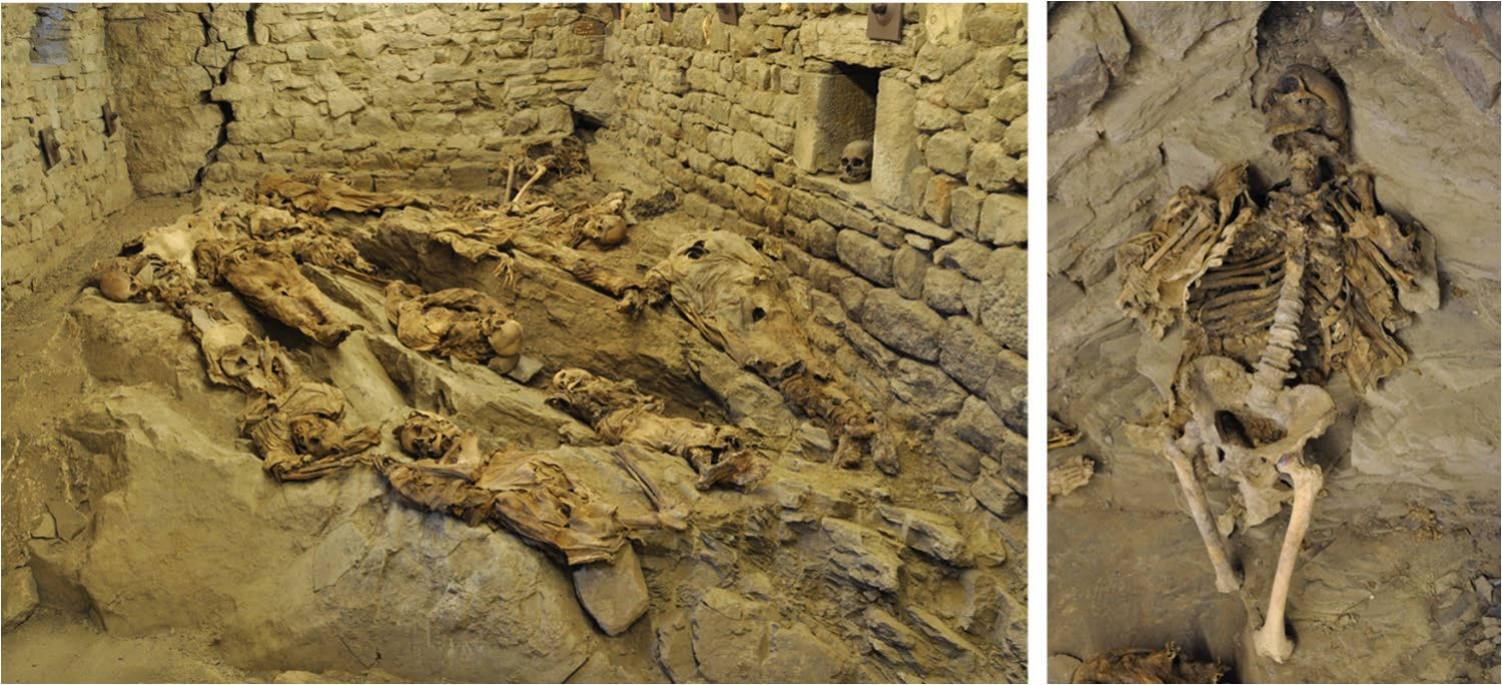
Left: crypt of the Roccapelago church, which houses twelve of the best preserved mummies. Right: SU23-id49 as it now appears inside the museum

During the excavation of the Church of the Conversion of San Paul, in Roccapelago (Modena, Northern Italy), a hidden crypt was discovered that contained over 400 individuals. Archaeological investigations were conducted between 2009 and 2011, under the direction of the Archaeological, Beautiful Arts and Landscape Superintendence of Bologna, Modena, Reggio Emilia and Ferrara. This crypt was used as a cemetery by the inhabitants of the small village of Roccapelago between the sixteenth and the eighteenth century [12]. The hidden crypt contained a large amount of human remains, including infants, subadults, and adults, many of which had undergone a usual process of decomposition. Numerous individuals, especially those from the most recent stratigraphic unit (SU23), were found relatively intact and well preserved. The presence of two small windows helped to maintain a dry and ventilated environment, which allowed for substantial mummification of their soft tissues. The analyses of the textiles suggest that the bodies were dressed in tunics and heavy socks and likely deposited within a shroud or sack. In agreement with the superintendency officials and a textile historian [13], twelve of the best-preserved and representative of the fashion of the time were not stripped and were studied only with minimally invasive methods (Fig. 1).

These mummies were entrusted to a museum, without any alteration and in accordance with the guidelines of the ICOM Code of Ethics for Museums for human remains [14]. Here, we study a polycystic lesion on the external condyle of a femur belonging to one of the twelve mummies.

## Materials and methods

**Fig. 2:**
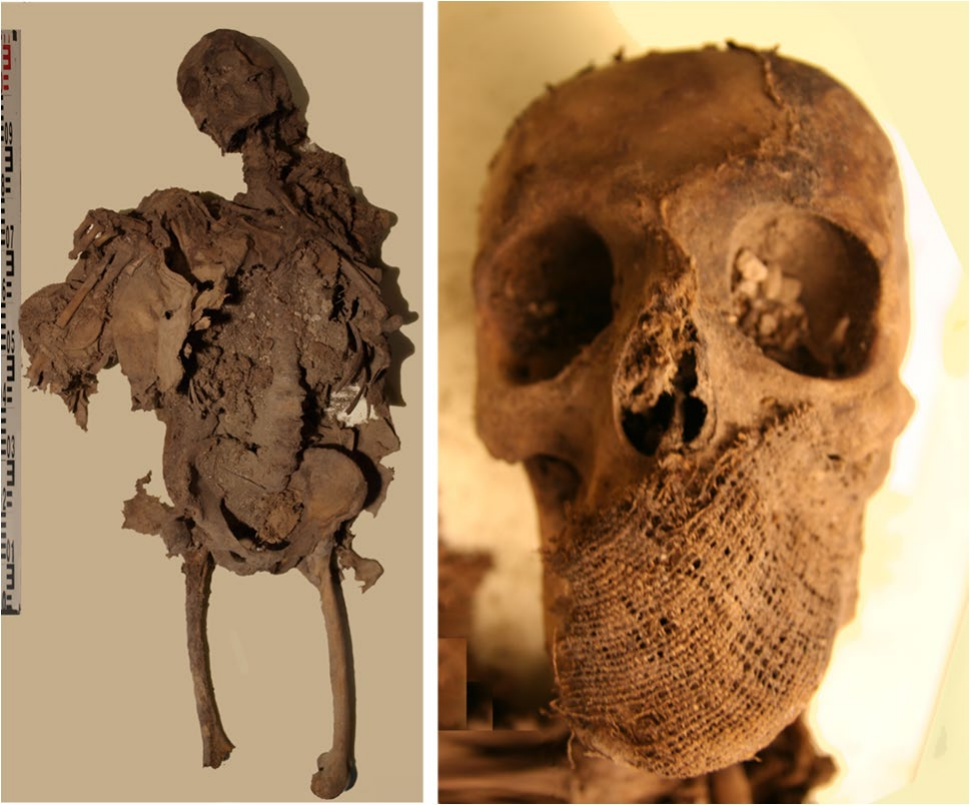
Left: SU23-id49 in the position in which the subject was found inside the crypt. Right: textile residue of the shroud adhered over the face, which covered most of the mummies of Roccapelago

The subject of this study is an adult individual (SU23-id49) whose skeleton is almost complete, with the exception of missing tibiae, fibulae, and bones of the feet. This individual is partially mummified, with mainly ligaments and tendons preserved, along with fragments of epidermis and muscle tissue from the chest, skull, and arms. The subject’s clothing, which was well preserved and remained largely intact in anatomical position, was dated to the late eighteenth century based on stratigraphy and archaeological findings (Fig. 2).

SU23-id49 was examined macroscopically where possible and subjected to radiological analysis. Sex estimation of the subject was evaluated using the sexually dimorphic traits of the os coxae, using Probabilistic Sex Diagnosis [15]. The skull was additionally used to assess sex using standard anthropological methods [16]. Due to the partial state of mummification of SU23-id49, it was not possible to apply multiple methods to determine the individual’s age at death. Primary centers of ossification were predominantly used. In cases where the centers were already fused, the degree of closure of the cranial sutures was used as a means of verification [17].

Anthropometric analysis was carried out using the digital image data and by calculating the pilastric index (relates to the degree of development of the linea aspera) and platymeric index (relates to the degree of flatness in the upper portion of the femoral diaphysis), to quantify diaphyseal shape and robusticity [16, 18] to assess the degree of muscle engagement and possible asymmetry in the long bones. A cortical thickness index of the mid-shaft of the femur (FEMCI) was further calculated according to the formula proposed by Mays et al. [19]. The FEMCI can be used as a proxy to evaluate potential cortical bone loss due to poor muscle engagement or degenerative diseases such as osteoporosis.

Finally, the general condition of health of individual SU23-id49 was first assessed by direct and radiological observation and then by comparing the pathologically affected areas with similar conditions published in Ortner’s seminal bone reference atlas [20].

Individual SU23-id49 was CT scanned at the Morgagni-Pierantoni city hospital (Forlì, Italy). High-quality images were generated using a MDCT 16 slices multi-detector scanner (General Electric Lightspeed System, Milwaukee, WI) with the following acquisition parameters: single helical scan, 1.25 slice thickness, reconstruction interval of 0.7 mm, and an energy of 120 kV and 140–300 mA [12, 21–23].

## Results

The position of the upper limbs of SU23-id49 when recovered was deemed to be affected by taphonomic phenomena. Based on archaeological records for this geographic region and time period, SU23-id49 was probably buried with the typical style of the hands crossed over the chest. Over time, this position likely moved to that of forearm flexion with the limbs resting parallel with the torso. Both persistent and labile joints have been preserved, which is an indication of SU23-id49’s primary deposition.

SU23-id49 was classified as an adult female who died between the age of 40 and 49 years old. This estimate was supported by information on the residues of the clothing and by the style of refined hemstitches and curls on the garment’s wrists and neck [13].

The mathematical regression calculated on the left femur provides a height of 152 cm, with the anthropometric indices demonstrating a marked asymmetry in the thigh bones. The results of the pilastric index calculated for the right femur show an absolute strong degree of expression at the mid-shaft (score > 120 = 129.4). By contrast, scores for the left femur reveal a weak degree of expression (score 100–109.9 = 101.3). The platymeric index, calculated at the proximal shaft display a degree of flatness for the right femur (score < 84.9 = 83.4) and a degree of roundness for the left femur (score 85–99.9 = 97.9). A marked asymmetry was further detected by the FEMCI, demonstrating differing cortical thickness indices for the right (55.37) and left (38.22) femurs.

**Fig. 3:**
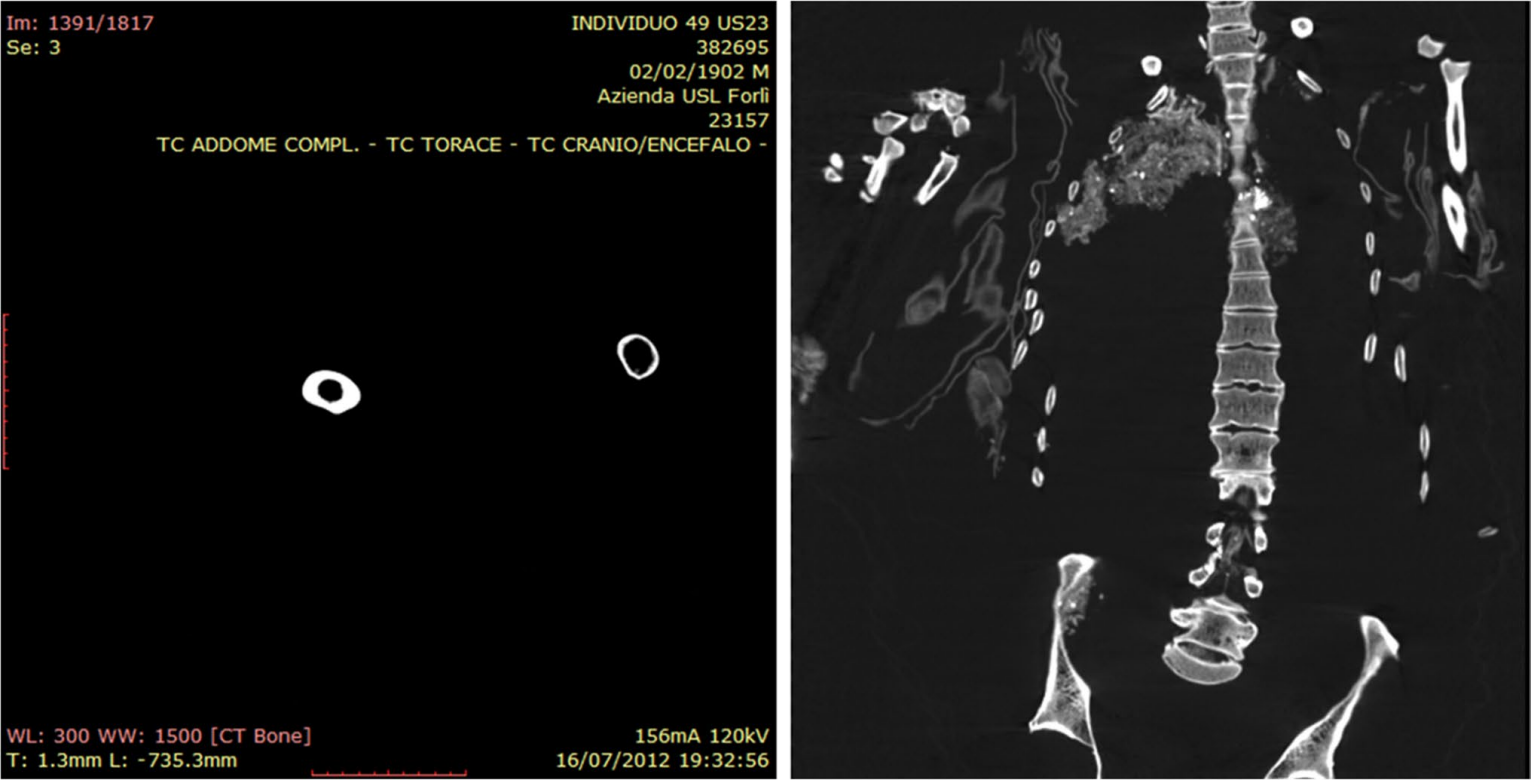
Left: cortical thickness differences of the femoral mid-shaft in axial view from the CT scan. Right: sagittal view of the vertebral column with numerous Schmorl’s nodes visible on the T8-T12 spinal segment

A paleopathological survey revealed several Schmorl’s nodes in the T8-T12 spinal segment and degenerative changes such as spondyloarthritis of the lumbar spine, with moderate osteophytosis (Fig. 3).

**Fig. 4:**
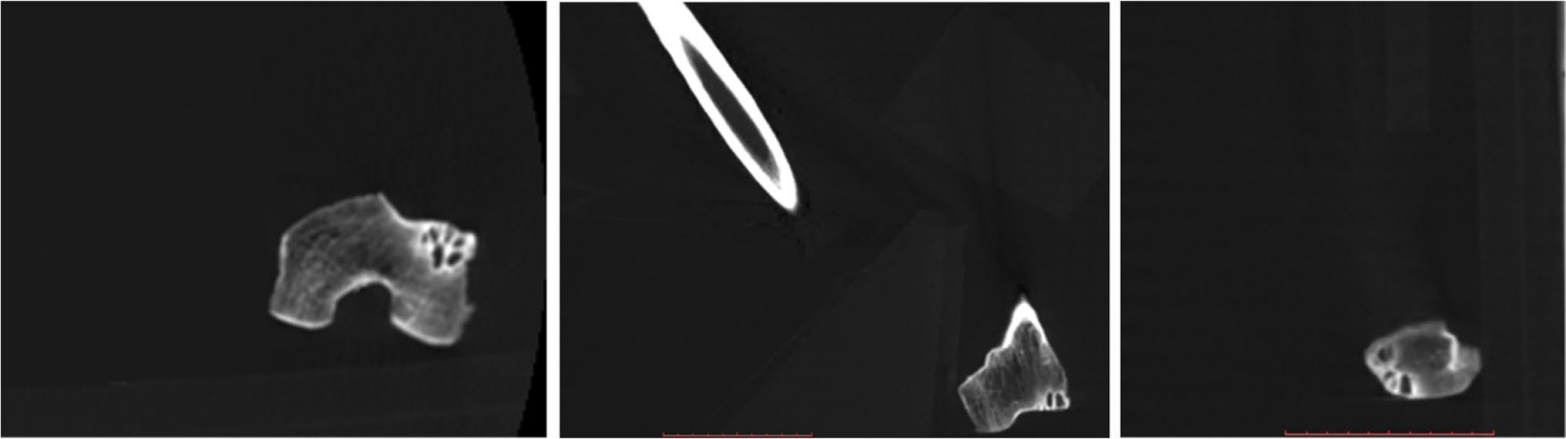
Left: distal epiphysis of the right femur in axial view showing the lytic lesion on the lateral condyle. Center: lateral condyle lesion in coronal view. Right: lateral condyle lesion in sagittal view

A cystic multiloculated lesion was additionally detected on the meta-epiphysis (i.e., the combined regions of the metaphysis and epiphysis, including the location of the growth plate) of the external condyle of the left femur, with absence of bone expansion, cortical osteolysis, and periosteal reaction. This latter particular lesion (Fig. 4) was used to formulate a differential diagnosis that was based solely on radiological features. Morphological features could not be evaluated using histology or other microscopy techniques [24] as biopsy tissue was not made available on account of the specimen conservation policy. Nevertheless, all entities included in standard histological differential diagnosis were easily ruled out by CT findings, given the peculiar characteristics of the lesion (location, shape of the lesion margins, absence of any type of bone reaction, age of the patient).

## Differential diagnosis

Lytic bone lesions may be caused by numerous differing conditions, including benign and malignant tumors, trauma, and infectious diseases.

Essential diagnostic criteria to GCT include a bone tumor with compatible radiographic imaging demonstrating an osteolytic circumscribed lesion involving the epiphysis, generally in a skeletally mature individual [1]. In long bones, it is typically eccentric (i.e., the lesion is not in the midline) and extends to the articular cartilage [1–3]. Trabeculation (the presence of trabeculae within the lesion) is common, ranging from fine to coarse, and are represented as endosteal ridging [24].

Aneurysmal bone cysts may be excluded in the diagnosis as they do not involve the epiphyses of bones. In addition, simple bone cysts do not often involve the epiphysis and the average age of affected patients is substantially younger (3–15 years old) than SU23-id49. NOF was easily ruled out, as it arises in the metaphysis of long bones in the lower extremities of skeletally immature individuals. Veritably, those lesions located in the epiphysis in skeletally mature individuals which were originally considered BFHs are now considered to represent bone GCTs, while those in the metaphysis are now agreed to represent NOFs. Malignant tumors were additionally excluded as the smooth edge and the sclerotic margin of the lesion favor a slowly growing benign disease process. Moreover, lytic metastases to the long bones have a predilection for the more-vascularized metaphyses, and the lack of any bone reaction further excludes osteosarcoma. Clear cell chondrosarcoma is a typical epiphyseal tumor of adulthood, but it develops preferentially in the femoral and humeral head, ribs, skull, spine, hands, and feet.

Therefore, a diagnosis of GCT is concluded with a high level of confidence. The examination of the characteristics of the lesion from CT images combined with considerations of the location of the lesion and the age of the individual are all in line with GCT. Moreover, probable pain due to claudication likely generated the asymmetry in the indices of the femur as the subject likely moved with a limp.

## Discussion and conclusion

GCT represents approximately 5% of all primary bone tumors and 20% of benign bone tumors. The peak incidence occurs between 20 and 45 years of age, with a slight predominance in females [1, 25]. These neoplasms are typical epiphyseal lesions affecting the ends of long bones (distal femur, proximal tibia, distal radius, and proximal humerus) in the mature skeleton [1–3]. A conventional GCT is a well-defined osteolytic lesion often eccentrically lying within the end of an asymmetrically expanded long bone [24]. The surrounding cortical bone is thinned or completely destroyed but is generally contained by a band of reactive periosteum. The subchondral bone plate can be focally eroded, but the articular cavity is seldom penetrated by the tumor.

The case of SU23-id49 clearly highlights a situation in which occupational activities have left an asymmetrical appearance to the bone structure. From the bone analyses, the lower limbs (relative to the upper limbs) show a different muscle engagement, which is much less intense, and with a significant loss of cortical bone mass in the left femur [19, 26]. The spine shows degenerative activity consistent with lesions arising from inflammatory processes and an uneven distribution of forces to the nucleus pulposus of the intervertebral disks, particularly in T8-12. It is therefore very likely that SU23-id49 lived with a chronic condition, which perhaps caused some functional limitation and required a redistribution of forces to the skeletal system. These findings are in accordance with clinical symptoms typically associated with GCT, including pain, swelling, and occasionally restricted movement [1].

A unique case of ancient GCT was described by Brothwell in the left femur of an adult subject from the Saxon site of Finglesham in Kent, UK [11]. He described a pronounced swelling approximately 120-mm long on the medial side of the shaft slightly above the distal condyles. The internal structure was characterized as comprising mainly of a thin cancellous inner zone surrounded by a bony shell varying in thickness. However, as the lesion was evaluated by macroscopic inspection alone, it may have been an incorrectly classified GCT (i.e., based on the misuse of giant cells, such as an aneurysmal bone cyst). It is important to note that the distal end of the femur is one of the most common sites of true GCTs, which is the affected location in individual SU23-id49.

NOF is a lesion strictly related to GCT and is classified in the same group by the World Health Organization (WHO) [8]. It represents a benign and generally self-limiting tumor of bone arising in skeletally immature individuals, with a peak incidence in the second decade of life. Related conditions, no longer accepted by WHO, include fibrous cortical defect (FCD), metaphyseal fibrous defect (MFD), and BFH [5, 6, 9, 10]. The vast majority of NOFs arise in the metaphysis of long bones of the lower extremities, especially around the knee and distal tibia [4, 8]. Their incidence is unknown, because most of these lesions lack specific symptoms and remain clinically silent. Radiologic surveys suggest that approximately 35% of otherwise normal children have occult NOFs, with a male/female ratio of 2.5:1 [2–4, 8]. These lesions usually heal and are completely remodeled during late childhood or adolescence. Larger tumors, however, may persist into adulthood and become painful due to microfractures and may even result in a pathological diaphyseal fracture [4, 8].

NOF is commonly observed in modern times, but represent a rare finding in paleopathology. Moreover, only a few similar cases have been reported in the paleopathological literature. For instance, Djuric et al. described a so-called FCD in the distal metaphyseal region of the femur in a 15–17-year-old skeleton of unknown sex found in the medieval cemetery of Stara Torina, Northern Serbia [27]. The lesion measured 15 mm in its largest diameter and consisted of an ovoid, smooth-edged eccentric cavitation located postero-medially in the insertion area of the adductor magnus muscle. Another report by Anderson detailed two MFD cases from a medieval cemetery in Norwich, UK, and mentioned an unpublished case of FCD found in a medieval cemetery in Canterbury, UK [28]. The Norwich skeletons were of subjects of undetermined sex, estimated at 12–15 and 15–17 years at death, and who both had cystic lesions in the knee region. The younger individual presented an ovoid smooth-edged eccentric cavitation on the postero-medial aspect of the distal femoral metaphysis, measuring 20 × 7 × 6 mm, and the older child had a similar defect in the lateral aspect of the proximal tibial metaphysis, measuring 20 × 15 × 7 mm. Both these lesions were multilocular with sclerotic borders. The location, morphology, and physiological age were consistent with the diagnosis of FCD/MFD. However, these cases should now be considered NOFs.

The apparent rarity of NOF and GCT lesions in the paleopathological record, in contrast to modern clinical findings, may be explained by the following factors. First, the actual prevalence of NOFs is undoubtedly under-represented in ancient skeletal remains because these lesions heal spontaneously leaving no visible skeletal deformity [28]. Thus, previous investigators recorded only visible pathological signs, naturally overlooking some of the associated discrete lesions (such as porotic lesions) accounted in their pathological surveys [27]. Second, and correspondingly, the lack of systematic radiological investigation of many ancient skeletal remains likely plays a large role in the rarity of ancient documented cases.

The importance of both X-ray examination and CT scanning as essential diagnostic tools in paleopathology has been extensively demonstrated [29–32]. Good-quality and high-resolution radiological scans and reconstructions performed by skilled professionals in radiology and pathology is of paramount importance in detecting and characterizing disease states and for establishing a correct diagnosis.
